# Progression of pediatric celiac disease from potential celiac disease to celiac disease: a retrospective cohort study

**DOI:** 10.1186/s12887-021-02625-z

**Published:** 2021-03-29

**Authors:** Shruti Sakhuja, Lori R. Holtz

**Affiliations:** Department of Pediatrics, Washington University School of Medicine, 660 S. Euclid, Campus, Box 8208, St. Louis, MO 63110 USA

**Keywords:** Celiac disease, Potential celiac disease, Pediatrics, Biopsy

## Abstract

**Background:**

A subset of patients with serology suggesting celiac disease have an initially negative biopsy but subsequently develop histopathologic celiac disease. Here we characterize patients with potential celiac disease who progress to celiac disease.

**Methods:**

We performed a retrospective analysis of children (0–18 years of age) with biopsy-confirmed celiac disease seen at St. Louis Children’s Hospital between 2013 and 2018.

**Results:**

Three hundred sixteen of 327 (96%) children with biopsy-confirmed celiac disease were diagnosed on initial biopsy. The 11 children with potential celiac disease who progressed to celiac disease had lower anti-tissue transglutaminase (anti-TTG IgA) concentrations (2.4 (1.6–5) X upper limit of normal (ULN) vs. 6.41 (3.4–10.5) X ULN) at time of first biopsy. Their median anti-TTG IgA concentrations rose from 2.4 (1.6–5) X ULN to 3.6 (3.1–9.2) X ULN between biopsies.

**Conclusions:**

Four percent of biopsy confirmed celiac patients initially had a negative biopsy, but later developed histopathologic celiac disease. This is likely an underestimate as no surveillance algorithm was in place. We recommend repeat assessment in children whose serology suggests celiac disease despite normal small bowel biopsy.

**Supplementary Information:**

The online version contains supplementary material available at 10.1186/s12887-021-02625-z.

## Introduction

Celiac disease is an autoimmune enteropathy triggered by gluten and its related proteins found in wheat, rye, and barley. Celiac disease causes inflammation and small intestinal damage of varying severity, and can have long-term adverse effects on growth and development [1].

The diagnosis of celiac disease is typically made with a combination of serological testing, including tissue transglutaminase (TTG IgA), anti-endomysial (EMA IgA), and deamidated gliadin (DGP) antibodies, as well as upper endoscopy with biopsy findings of villous blunting, villous atrophy, or crypt hyperplasia [1, 2]. A subset of patients in the population have positive celiac serology, but have normal duodenal villous architecture on endoscopy [3, 4]. This situation has been termed potential celiac disease [3–5]. Of particular interest, are the patients with positive serology and an initially normal biopsy, who develop villous atrophy on a subsequent biopsy.

Previous studies have suggested that the amount of intraepithelial lymphocytosis (IEL) on initial biopsy [6, 7], the presence of EMA IgA [8, 9], or the presence of certain HLA-DR and DQ genotyping [10–12] can serve as good markers for progression to celiac disease. Our study aims to additionally characterize patients with potential celiac disease who progress to celiac disease by variables such as age, sex, race, comorbidities, and laboratory values. Using these predictive characteristics, we hope for more accurate identification of patients with initially elevated celiac serology who will then go on to develop disease, and thus create a new framework for the diagnosis and management of the disease.

## Methods

### Patients and data collection

This study was approved by the Washington University Human Research Protection Office. We conducted a retrospective chart review of children (< 18 years old) treated by the Division of Pediatric Gastroenterology, Hepatology, and Nutrition at St. Louis Children’s Hospital (SLCH) between November 2013 and May 2018. St. Louis Children’s Hospital is a tertiary, free-standing 402 bed children’s hospital serving a six state region. Our study’s inclusion criteria were relevant ICD-9 and 10 codes (579.0, K90.0, R76.0, R76.8, R76.9, R89.4, 795.79), anti-TTG IgA concentration available at time of evaluation, and at least one upper endoscopy with four biopsies taken from the second portion of the duodenum and two from the bulb [1]. At SLCH modified Marsh scores and CD3 staining were not universally reported by the pathologists. A biopsy was defined as positive for celiac disease if it demonstrated villous blunting or atrophy or crypt hyperplasia. It was considered negative if the villi appeared normal or there was intraepithelial lymphocytosis characterized as “mild” in the pathology report.

All information was extracted from SLCH’s electronic medical record. Because laboratories have differing normal ranges for circulating anti-TTG IgA concentrations based on assay kit, values were described in relation to the upper limit of normal (times the upper limit of normal, X ULN) of the test as used in previous studies [13].

The World Health Organization anthropometric calculator was used to obtain height-for-age Z-score (HAZ) and weight-for-age Z-score (WAZ), as well as percentiles.

### Statistical methods

Data collected in this study were analyzed using Prism (v8.4.1) statistical software. The Mann-Whitney test was used to determine whether there was a statistical difference between continuous variables such as TTG concentrations. The Fisher Exact test was used to calculate *p* values for presence or absence of type I diabetes, hypothyroidism, and trisomy 21 in the celiac disease and potential celiac disease groups. All *p*-values were two tailed. All data are presented as median (interquartile range) or number (percentile), as appropriate.

## Results

We identified 327 patients with biopsy-confirmed celiac disease at St. Louis Children’s Hospital during the study interval. Children with initially potential celiac disease who progressed to celiac disease were referred for celiac evaluation predominantly for other autoimmune conditions (i.e. type I diabetes mellitus, hypothyroidism), trisomy 21, and symptoms consistent with celiac disease including abdominal pain, constipation, diarrhea, and poor growth. Children with celiac disease confirmed on the initial biopsy were additionally referred because of family history of celiac disease, and/or other gastrointestinal illnesses (i.e. eosinophilic esophagitis, inflammatory bowel disease). Serological testing in the combined celiac disease and potential celiac disease who progressed to celiac disease groups consisted of anti-TTG IgA (100%), anti-EMA IgA (18%), and anti-DGP IgG (8%). These laboratory values were obtained at either St. Louis Children’s Hospital (78%), or other laboratories in our region (22%).

**Fig. 1:**
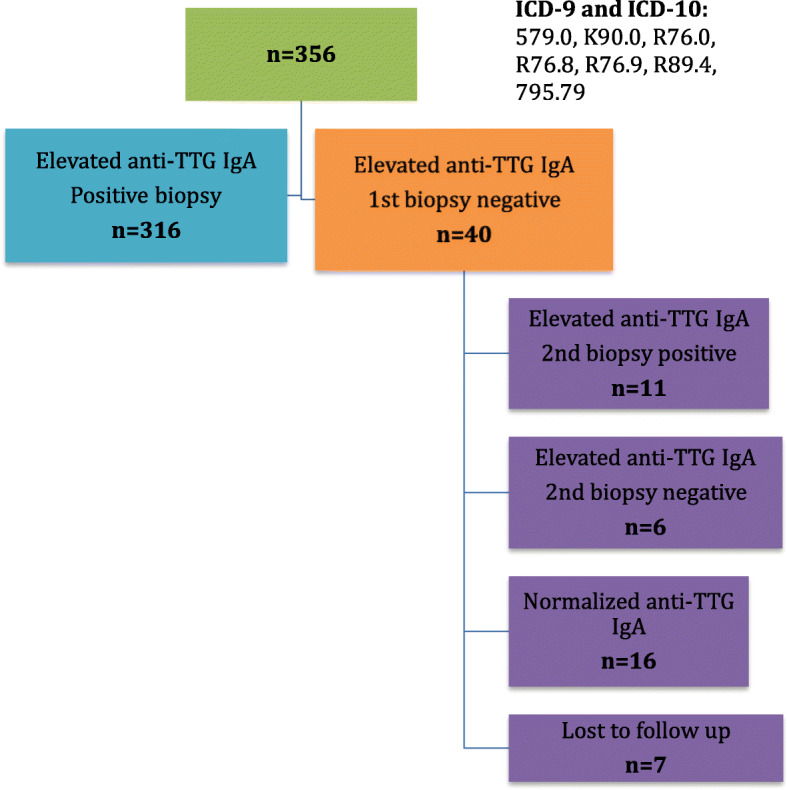
Overview of the distribution of patients meeting inclusion criteria

We also identified 29 additional patients who had initially elevated anti-TTG IgA concentrations with a normal first biopsy, but who underwent a second biopsy that was negative and therefore were not diagnosed with celiac disease (*n* = 6, 2%), had anti-TTG IgA concentration that normalized on repeat testing and therefore did not undergo repeat endoscopy (*n* = 16, 4%), or were lost to follow up (*n* = 7, 2%) (Fig. 1).

Of the 327 patients with biopsy-confirmed celiac disease, 316 (96%) had a positive initial biopsy. Eleven (4%) had a negative initial biopsy. Eight of these 11 (73%) had evidence of mild IEL on biopsy and 3 had no histological abnormalities. These 11 patients remained on a gluten-containing diet while their celiac serology was serially monitored, and, on repeat biopsy, had histologic features of celiac disease.

**Fig. 2:**
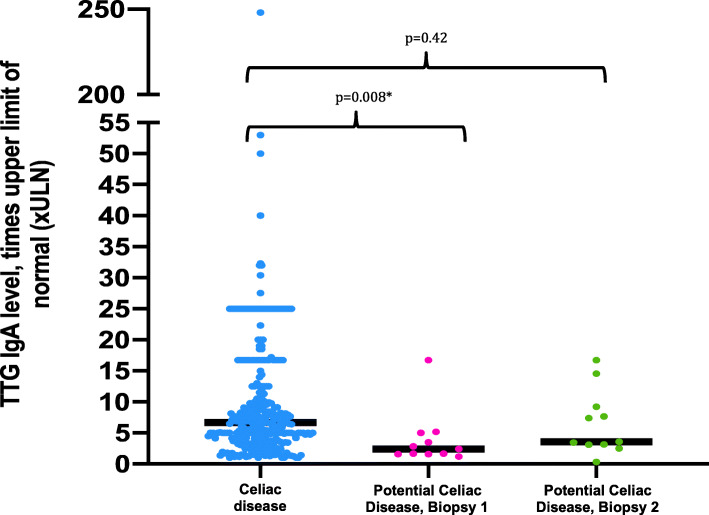
Anti-TTG IgA levels in patients with celiac disease on initial biopsy vs. potential celiac disease (initial biopsy without disease and second biopsy with disease). Statistical significance determined using Mann Whitney test

Of the 316 patients with a positive initial biopsy, 220 (70%) patients were female (Table 1). The median anti-TTG IgA concentration for patients with celiac disease confirmed on initial biopsy was 6.41 (3.4–10.5) X ULN (Fig. 2). Notably, only 104 (33%) of the patients who had abnormal anti-TTG IgA concentrations and a positive biopsy had anti-TTG IgA concentrations > 10 X ULN. Furthermore, 66 (21%) patients even had an anti-TTG IgA concentration < 3 X ULN with a positive biopsy.

**Table 1:** Demographic data and characteristics of study participants *n (%)*

	Celiac disease confirmed on initial biopsy, ***n*** = 316 (96)	Potential celiac disease, time of initial biopsy, ***n*** = 11 (4)	Celiac disease vs potential celiac disease initial biopsy *p* values	Potential celiac disease, time of second biopsy, ***n*** = 11 (4)	Celiac disease vs potential celiac disease second biopsy *p* values
**Median age at diagnosis, yr (IQR)**	10 (6–13)			13 (9–15)	*p* = 0.03*
**Female**	220 (70)	8 (73)	*p* = 1.00	8 (73)	*p* = 1.00
**Caucasian**	307 (97)	11 (100)	*p* = 1.00	11 (100)	*p* = 1.00
**Median HAZ (IQR)**	(−)0.3 ((−)1.15–0.6)	(−)0.15 ((−)0.92–0.44)	*p* = 0.76	0.03 ((−)0.76–0.20)	*p* = 0.71
**Median WAZ (IQR)**	(−)0.22 ((−)0.925–0.51)	(−)0.04 ((−)0.78–0.47)	*p* = 0.93	0.39 ((−)0.62–0.85)	*p* = 0.46
**Median BMI percentile (IQR)**	58 (27–84)	48 (41–75)	*p* = 0.89	59 (33–87)	*p* = 0.63
**Median hemoglobin concentration (IQR)**	12.8 (11.9–13.6)	13.7 (12.7–14.9)	*p* = 0.10	13.8 (11.9–14.4)	*p* = 0.25
**Median MCV (IQR)**	82.6 (78.8–85.3)	87.4 (81.3–90.8)	*p* = 0.03*	83.3 (81.5–89.3)	*p* = 0.21
**Family history of celiac disease**	55 (17)	0 (0)	*p* = 0.22	0 (0)	*p* = 0.22

Of the 11 children with potential celiac disease who then progressed to celiac disease, 8 (73%) were female (Table 1). Each of these 11 patients had abnormal anti-TTG IgA concentrations at the time of initial intestinal biopsy, and 10 had persistently elevated anti-TTG IgA at the time of the second biopsy. All reported remaining on a gluten-containing diet. The median interval between the first and second biopsies was 14 (10–30) months. The median anti-TTG IgA concentration for these 11 patients at time of initial biopsy rose from 2.4 X ULN (1.6–5 X ULN) to 3.6 X ULN (3.1–9.2 X ULN) (*p* = 0.11) between biopsies (Fig. 2).

The anti-TTG IgA concentrations between the two groups at time of initial biopsy was significantly lower in the potential celiac disease group (*p* = 0.008) (Fig. 2). However, there was no statistically significant difference (*p* = 0.42) between anti-TTG IgA concentrations of the celiac disease group and the potential celiac disease group at time of subsequent diagnosis.

The median age at diagnosis of celiac disease in those patients diagnosed on the initial biopsy was 10 (6–13) years; the median age at diagnosis was 13 (9–15) years in patients with potential celiac disease who progressed to celiac disease (*p* = 0.03) (Table 1).

Patients with celiac disease confirmed on the initial biopsy had a median weight percentile of 49 (16–75) and median height percentile of 58 (30–87). The median weight percentile of patients with potential celiac disease was 50 (25–70) at time of initial biopsy, and 55 (40–80) at time of second biopsy when the diagnosis of celiac disease was made. The median height percentile of patients with potential celiac disease was 54 (34–82) at time of initial biopsy, and 60 (22–67) at time of diagnosis with second biopsy. There was no statistically significant difference in the weight and height percentiles between the celiac disease and potential celiac disease groups at time of either the initial or second biopsy.

The median height-for-age Z-score (HAZ score) for patients with celiac disease was − 0.3 (− 1.15–0.6), compared to − 0.15 (− 0.92–0.44) for patients with potential celiac disease at the time of the first biopsy (*p* = 0.76), and 0.03 (− 0.76–0.20) at the time of the second biopsy (*p* = 0.71). The median weight-for-age Z-score (WAZ score) for patients with celiac disease was − 0.22 (− 0.925–0.51), compared to − 0.04 (− 0.78–0.47) for patients with potential celiac disease at time of first biopsy (*p* = 0.93), and 0.39 (− 0.62–0.85) at time of second biopsy (*p* = 0.46). There was again no statistically significant difference in the HAZ and WAZ scores between the celiac disease and potential celiac disease groups at time of first or second biopsy (Table 1).

Of the 316 patients diagnosed with celiac disease on the initial biopsy, 146 had a hemoglobin concentration obtained at time of diagnosis. Their median hemoglobin concentration was 12.8 g/dl (11.9–13.6) and median mean corpuscular volume (MCV) was 82.6 fL (78.8–85.3) (Table 1). Of the 11 patients with potential celiac disease who progressed to celiac disease, 7 had a hemoglobin concentration available at time of first and second biopsy. At time of first biopsy, their median hemoglobin concentration was 13.7 g/dl (12.7–14.9), and median MCV was 87.4 fL (81.3–90.8). At time of second biopsy, the median hemoglobin concentration was 13.8 g/dl (11.9–14.4), and median MCV was 83.3 fL (81.5–89.3). There was no statistically significant difference in the hemoglobin concentrations or MCV between both groups (Table 1).

**Fig. 3:**
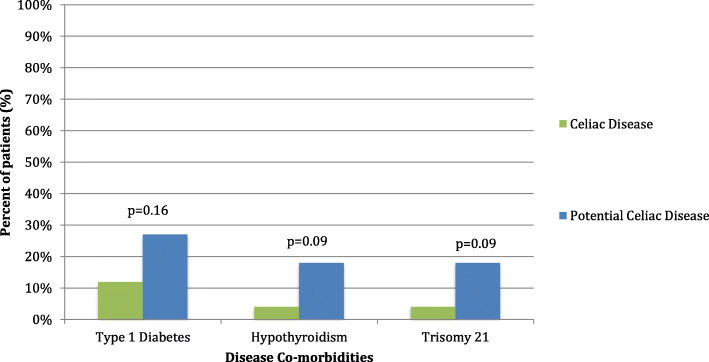
Disease co-morbidities in patients with celiac disease and potential celiac disease who progress to celiac disease on second biopsy. Statistical significance determined using Fisher Exact test

Among the patient diagnosed with celiac disease on the initial biopsy, 40 (12%) patients had been diagnosed with type I diabetes mellitus (T1DM), 13 (4%) with hypothyroidism, and 13 (4%) with trisomy 21. In those patients with potential celiac disease which progressed to celiac disease, 3 (27%) patients had T1DM, 2 (18%) had hypothyroidism, and 2 (18%) had trisomy 21 (Fig. 3). There was no statistically significant difference in prevalence of T1DM (*p* = 0.16), hypothyroidism (*p* = 0.09), or trisomy 21 (*p* = 0.09) between the groups. However in the aggregate, 61 (19%) of patients with celiac disease on the initial biopsy had been diagnosed with at least one of these three co-morbidities, versus 5 (45%) of the 11 patients in the potential celiac disease group (*p* = 0.05). Additionally, five unique patients in the celiac disease group had other inflammatory disorders of the digestive system: two (< 1%) had concomitant ulcerative colitis, and 3 (1%) had eosinophilic esophagitis.

## Discussion

Prior studies of potential celiac disease [3, 4, 6] suggest that intraepithelial lymphocytosis on initial biopsy may be an early presentation of celiac disease. Auricchio et al. [7] also suggested that both quantity and type of intraepithelial lymphocytes may predict who subsequently develops celiac disease. In our study, 8 of our 11 patients (73%) with potential celiac disease had evidence of non-specific inflammation with IEL, but their IEL was described as mild on the biopsy, calling into question the predictive value of this semi-quantitative measurement.

A striking finding was the poor performance of using a cutoff point of 10 X ULN to predict a diagnosis of celiac disease before (or in lieu of) performing a biopsy in this North American population. Indeed, 67% of the patients with definite diagnosis of celiac disease on biopsy had anti-TTG values less than this cutoff point, and 21% had values less than 3 X ULN. Therefore, it may be worthwhile to pursue duodenal biopsy even in patients with anti-TTG values less than 3 X ULN.

Anti-EMA antibody has previously been reported as a good predictor for the subsequent diagnosis of celiac disease in patients whose initial biopsy is negative [8, 9]. In our study, 5 of the 11 patients with potential celiac disease had an anti-EMA obtained. In these 5 patients, anti-EMA was indeed correspondingly elevated at both time of initial biopsy and second biopsy (see Supplementary Table 1), thereby supporting the potential use of anti-EMA as a predictor for subsequent diagnosis of celiac disease when the initial biopsy is negative. We further demonstrated that the degree of circulating anti-TTG IgA elevation also appears to correlate with the histopathologic diagnosis of celiac disease as patients in both our potential celiac disease and celiac disease cohorts had a higher relative anti-TTG IgA concentration at the time when celiac disease was diagnosed. However, there is insufficient overlap between groups to make this value useful as a clinical tool.

The majority of patients within both cohorts were non-Hispanic white, as supported in the literature [12, 14]. The female predominance resembles sex ratios of other series of children with celiac disease [12, 15, 16]. However, there was a statistically significant difference in age at time of diagnosis between the two cohorts, and the potential celiac disease group had a higher proportion of co-morbidities which prompted screening. It is possible that such lead time bias contributes to initially negative biopsies, and these patients should especially be considered to be at risk of subsequent diagnosis if their initial biopsy is normal. The association of sex and presence of co-morbidities with subsequent diagnosis of celiac disease has been replicated in other studies on the progression of celiac disease [17]. There was no difference in stature, weight, or hemoglobin concentrations between the two cohorts, and in fact most patients in both cohorts had normal growth and normal hemoglobin concentrations, similar to recent series [18–20]. We do note the previously described challenges in interpretation of histopathology in celiac disease [21, 22] as a limitation of our study, which relied on one pathologist’s review of biopsies. This known variability underlies inclusion of serology and history in a “synoptic” assessment [23]. Additionally, we were unable to provide Marsh scores or quantification of intraepithelial lymphocytes on biopsy, and instead relied on qualitative descriptors of the biopsy findings per pathology reports. Lastly, because of the pragmatic nature of our study anti-TTG IgA concentrations were obtained for clinical purposes and were therefore performed using different assays, and potentially useful markers such as anti-EMA and HLA genotyping were not uniformly ordered.

We also note that this study highlights a potential shortcoming of the current celiac disease nomenclature. The personalized natural history of celiac disease is now recognized, but terms that embody the stages of progression are lacking. While potential celiac disease denotes patients characterized by positive serological markers and negative biopsies, there is no term which accurately defines the subsets of these patients whose serology normalizes vs. those whose biopsies become positive. We recognize that these classifications can only be made in retrospect, but advocate for nosologic flexibility to describe an entity in the celiac spectrum that encompasses progression as well as regression.

## Conclusion

Based on our study findings and previous work [3, 4, 6–9, 17], a subset of children with elevated celiac serologies are at risk of subsequently being diagnosed with this disorder. The percentage we report, 4%, is likely to underestimate this risk, because we did not systematically follow all patients with normal biopsies in this retrospective review. This study highlights the importance of close monitoring of patients who present to providers with concern for celiac disease and are found to have elevated anti-TTG IgA concentrations, even if their initial biopsy was negative. Additionally, our study suggests that even if the anti-TTG IgA levels are only modestly elevated, it may still be worthwhile to obtain a small bowel biopsy as some of these patients have evidence of villous atrophy. These data suggest that changes in diagnostic protocols and disease categorization might be warranted to optimize the management of children with suspected celiac disease.

## Supplementary Information

**Additional file 1: Table S1.** Demographic Data and Lab Results of Potential Celiac Patients. Demographic data, celiac serologies, and biopsy results of patients with potential celiac disease who progress to celiac disease on second biopsy.

## Data Availability

The datasets used and/or analyzed during the current study are available from the corresponding author on reasonable request.

## Abbreviations

SLCH: St. Louis Children’s Hospital
IQR: Interquartile range
ULN: Upper limit of normal
TTG IgA: Tissue transglutaminase
EMA IgA: Anti-endomysial
DGP: Deamidated gliadin
IEL: Intraepithelial lymphocytosis
HAZ: Height-for-age Z score
WAZ: Weight-for-age Z score
MCV: Mean corpuscular volume
T1DM: Type 1 diabetes mellitus
