# Supplementary material for: Progression of pediatric celiac disease from potential celiac disease to celiac disease: a retrospective cohort study
Source: BMC Pediatr. 2021 Mar 29;21:149. doi: 10.1186/s12887-021-02625-z (PMC8006356; doi:10.1186/s12887-021-02625-z)
Supplement: Supplementary file 1 — Additional file 1: Table S1. Demographic Data and Lab Results of Potential Celiac Patients. Demographic data, celiac serologies, and biopsy results of patients with potential celiac disease who progress to celiac disease on second biopsy. [file 12887_2021_2625_MOESM1_ESM.pdf]

Supplementary Table 1. Demographic Data and Lab Results of Potential Celiac Patients

ICD 9/10	Gender	Race	Age at Encounter1	Age at Encounter2	Weight percentile at Encounter1	Weight percentile at Encounter 2	Height percentile at Encounter1	Height percentile at Encounter2	BMI (kg/m2) at Encounter1	BMI (kg/m2) at Encounter2	TTG IgA X ULN at Encounter1	TTG IgA X ULN at Encounter2	Endomysial antibody	Biopsy #1 Result	Biopsy #2 Result	Inter-biopsy interval (months)
795.79	F	Caucasian	8	9	80%	80%	99%	99%	16	16	2.85	3.45	positive	mild lymphocytosis in duodenum without villous blunting	duodenitis with lymphocytosis and partial villous blunting	14
579.00	F	Caucasian	12	15	30%	30%	54%	60%	17.5	unknown	1.2	3.59	not obtained	focal mild lymphocytosis in duodenum	duodenitis with focal mild lymphocytosis and partial villous blunting	30
564.00	F	Caucasian	8	16	15%	95%	6%	6%	unknown	34.5	1.66	9.2	not obtained	mild, non-specific findings in duodenum	villous blunting with marked intraepithelial lymphocytosis in duodenum, plasmocytosis, prominent lymphoid follicle	91
R11.0	F	Caucasian	13	14	65%	65%	60%	60%	unknown	20.78	3.49	3.08	positive	no histopathologic abnormality in duodenum	subtotal to total villous atrophy with intramucosal lymphocytes and hypercellular lamina propria with plasma cells	9
R76.8	F	Caucasian	7	9	75%	80%	47%	47%	17	19.1	1.68	14.55	positive	increased intraepithelial lymphocytes in duodenum	focal villous blunting with increased lymphocytes in superficial epithelium	26
R10.84	F	Caucasian	7	7	28%	55%	54%	61%	15	14	5	3.16	not obtained	focal active duodenitis with preserved villous architecture	patchy, multifocal villous blunting with intraepithelial lymphocytosis, increased lymphoplastic component in lamina propria, focal intraepithelial neutrophils	6
K59.00	F	Caucasian	16	17	20%	18%	17%	20%	20	21	2.39	0.33	not obtained	mild increase in acute and chronic inflammation	villous blunting with intraepithelial lymphocytosis, focal periglandular acute inflammation	10
	F	Caucasian	9	10	70%	55%	86%	79%	16	15.8	>16.7	>16.7	positive	normal	villous blunting, intraepithelial lymphocytes, crypt hyperplasia, focal acute inflammation	11
795.79	M	Caucasian	7	12	70%	75%	30%	29%	17	23	1.58	2.5	not obtained	focal mild lamina propria acute inflammation in duodenum	mild villous blunting, mild focal lamina propria acute inflammation	42
795.79	M	Caucasian	11	13	50%	50%	69%	60%	19	20	1.59	7.4	positive	mixed villous architecture	villous blunting with increased intraepithelial lymphocytes	18
R76.8	M	Caucasian	14	15	25%	40%	unknown	unknown	unknown	unknown	5.2	7.65	not obtained	preserved villous architecture with focally increased intraepithelial lymphocytes	villous blunting in duodenum, increased intraepithelial lymphocytosis	10
